# Molecular basis of autism spectrum disorders

**DOI:** 10.1007/s11033-025-10604-1

**Published:** 2025-05-28

**Authors:** Raina Desai, Sumanjali Reddy, Michael Truong, Finosh G. Thankam

**Affiliations:** Department of Translational Research, College of Osteopathic Medicine of the Pacific, Western University of Health Sciences, 309 E. Second Street, Pomona, CA 91766-1854 USA

**Keywords:** Autism spectrum disorder, Molecular pathways, Neuroinflammation, Behavioral changes, ASD diagnosis

## Abstract

Autism is a prevalent neurological disorder that affects the development of children worldwide. With the advent of genome wide association studies and whole exome sequencing methods, associated genes and molecular pathways that lead to the development of autism spectrum disorder (ASD) have garnered recent attention. However, the etiology of ASD is still rudimentary and poorly understood. Thus, understanding the specific molecular pathways that contribute to autism aids in its clinical diagnosis, management, and treatment. Currently, the genes involved are expansive and affect many molecular pathways. These include transcriptional and translational regulation, proteostasis, cytoskeletal organization, synaptic development and plasticity, and autophagy. Additionally, there are a multitude of factors involved in ASD pathogenesis, including neuroinflammation, signaling defects, and vitamin deficiencies. This review aims to provide a comprehensive understanding regarding the genes and underlying molecular mechanisms that contribute to ASD and the translational therapies for the management of ASD.

**Graphical abstract:**

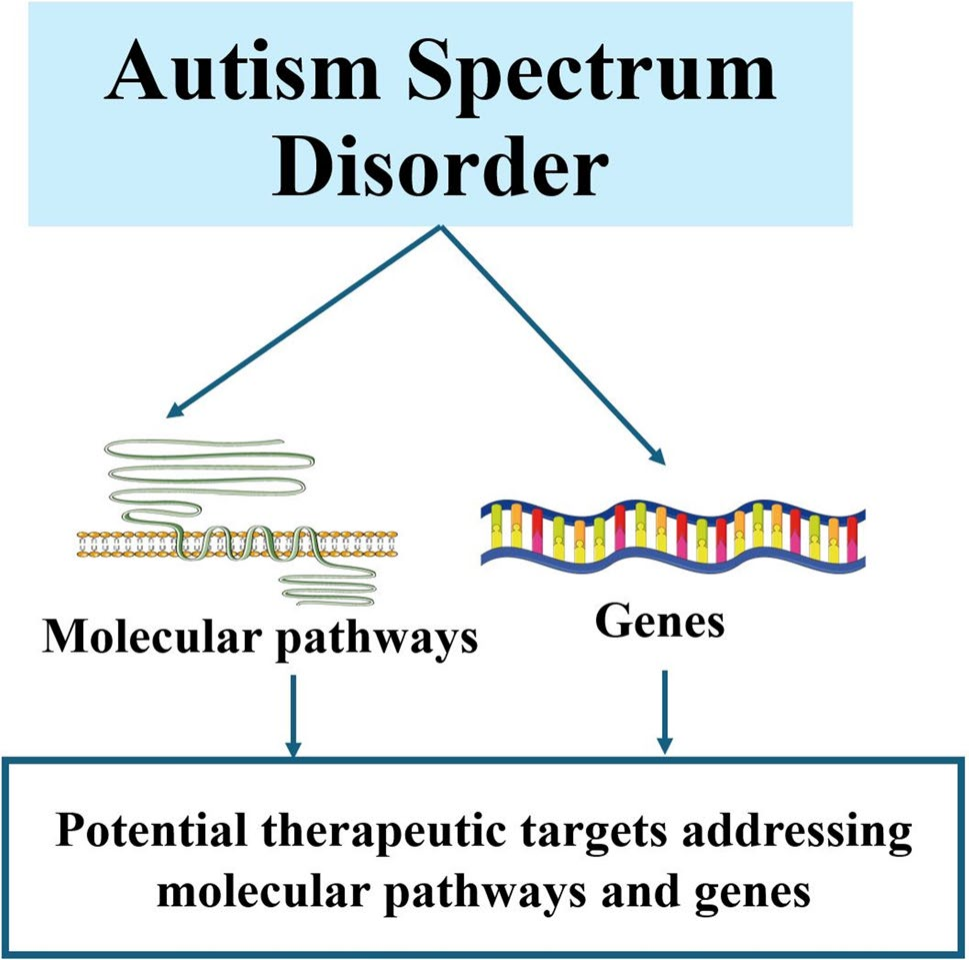

## Introduction

**Fig. 1:**
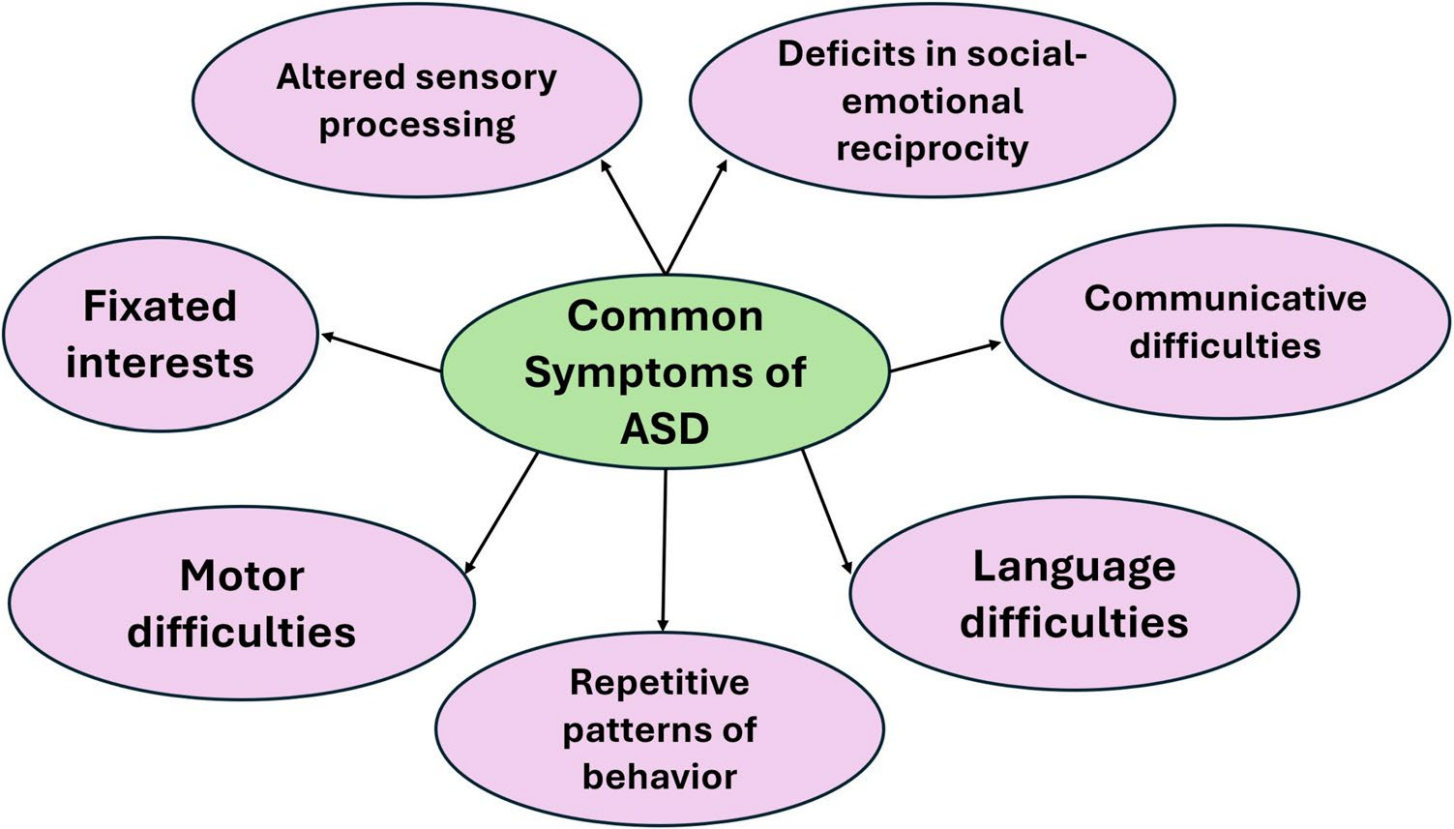
Common Symptoms of ASD. This figure illustrates common motor and sensory deficits associated with ASD pathogenesis [2, 3]. This figure was created using Google Docs

Autism, or autism spectrum disorder (ASD), is a neurological disorder that affects almost 3% of 8-year-olds across the United States [1]. ASD is associated with deficits in social communication with restricted and repetitive behaviors [2]. According to the DSM-5-TR, a child with ASD exhibits persistent deficits in “social communication and interaction across multiple contexts, as manifested by all of the following”: persistent deficits in social-emotional reciprocity, nonverbal communicative behaviors in social interaction, and developing, maintaining, and understanding relationships. In addition, diagnosis includes restricted, repetitive patterns of behavior, interests, or activities, such as stereotyped or repetitive motor movements, insistence on sameness, fixated interests that are abnormal in intensity or focus, and hyper- or hypo-reactivity to sensory input or unusual interest in sensory aspects of the environment [3] (Fig. 1). In the past decade, research efforts on ASD have progressed, which may contribute to its increasing prevalence worldwide [4]. However, establishing clear etiology has proven to be challenging because of genetic and environmental factors [5]. A number of genes contribute to the development of ASD. The molecular pathways of these genes include transcriptional regulation, proteostasis, cytoskeletal organization, and synaptic development and plasticity. Ultimately, research continues to reveal factors that correlate with ASD risk; however, casual determinations are limited [6]. This article outlines our current understanding regarding the specific genes and underlying molecular pathways associated with ASD.

### Etiology of ASD

Multiple subtypes of ASD exist, which include Asperger’s syndrome, Rett’s Syndrome, Childhood disintegrative disorder, Kanner’s Syndrome, and Pervasive Developmental Disorder-Not Otherwise Specified (PDD-NOS). These subtypes allow for the further classification of the condition. In the DSM-IV, before the term “autism spectrum disorder (ASD)” was established, these subtypes were regarded as five separate conditions. With the advent of the DSM-V, these formerly separate conditions have been brought under one umbrella term: ASD. In addition, ASD is a neurological disorder with two etiological forms: secondary and idiopathic. However, ASD arising from a specific cause only accounts for approximately 15% of cases, with 5% of cases being monogenetic [7]. This is termed as “secondary” ASD. In idiopathic ASD, which accounts for approximately 85% of all ASD cases, the cause is unknown [7].

Genome wide association studies and whole exome sequencing methods have advanced our understanding regarding the specific genes contributing to ASD [8]. The molecular pathology is attributed to the genes and proteins involved in brain development [9], neuroinflammatory pathways [10], and normal processes of DNA replication and expression [9, 11, 12]. Documented causes of secondary ASD include tuberous sclerosis, Down Syndrome, Fragile X syndrome, and some congenital infections including cytomegalovirus [13–15].

While genetics play a significant role in the development of ASD, the subsequent phenotypic expression is more variable. Manoli et al. demonstrated that alterations in ASD risk genes lead to a wide range of observable phenotypes, behaviors, and perceptions. Thus, defining the genes responsible for ASD pathology vary from patient to patient [16].

### Molecular biology of ASD

**Fig. 2:**
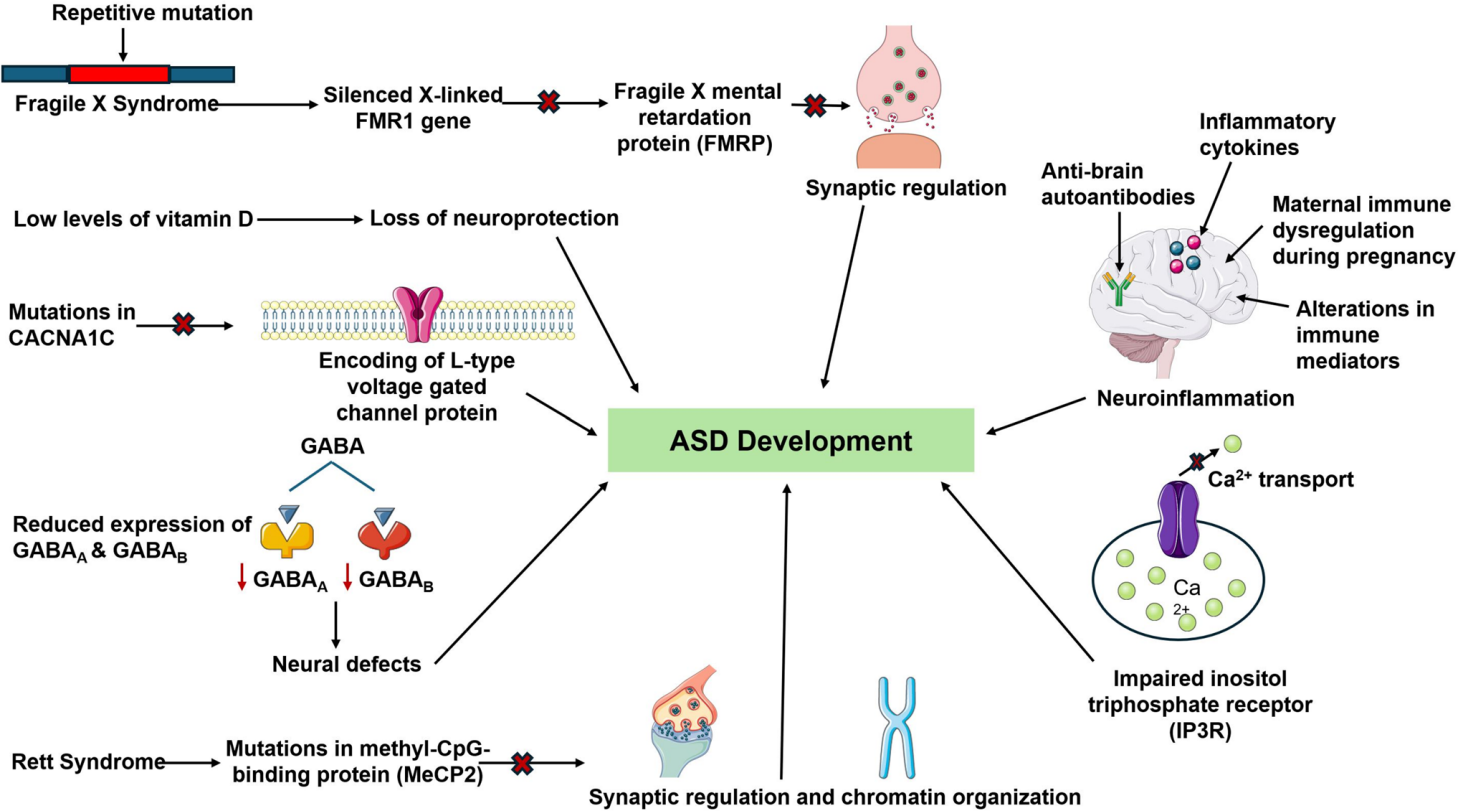
Overview of the Potential Factors Underlying Autism Spectrum Disorder (ASD). This illustration demonstrates the potential factors contributing to the development of ASD, such as signaling defects (i.e. GABA, neuronal calcium), neuroinflammation, vitamin D deficiency, Fragile X Syndrome (FXS), and Rett Syndrome. These factors may be involved in the underlying molecular pathways and disease process of ASD [17–29]. This figure was created using Microsoft PowerPoint and Smart Servier Medical Art

The pathogenesis of ASD is complex, involving multiple underlying molecular mechanisms. Recent evidence suggests that a multitude of factors including signaling defects, immunity, vitamin D deficiency, disruptions in metabolic pathways, Fragile X syndrome (FXS), and Rett syndrome are involved in ASD pathology and the associated symptoms (Fig. 2). Gamma amino butyric acid (GABA) is a crucial inhibitory neurotransmitter responsible for neural network formation. Dysfunctions in the GABAergic inhibitory system is an established mechanism associated with neural defects in ASD [17]. Reduced expression of GABA receptor subunits, specifically GABA_A_ and GABA_B_, and decreased differentiation and migration of GABAergic neurons underlie sensory processing symptoms and abnormalities observed in ASD [17, 18]. Further, Johnson et al. reported a correlation between GABAergic/glutamatergic imbalance and social impairment in autistic males, suggesting that both impaired inhibitory and excitatory signaling play a role in ASD pathogenesis. However, there is conflicting evidence regarding the increased excitation to inhibition ratio as an underlying factor of hyperexcitability in ASD. A seminal study proposed that the elevated excitation-inhibition ratio reflects the neuronal circuits restoring a normal cortical firing rates to maintain the homeostasis. This suggests that the altered excitation-inhibition ratio observed in ASD plays a role in synaptic homeostasis rather than contributing to hyperexcitability [19]. Calcium (Ca^2+^) is a critical second messenger involved in numerous molecular mechanisms in the synaptic transmission between neurons and is responsible for triggering neurotransmitter release at the presynaptic nerve terminal via Ca^2+^ influx [20]. Impaired neuronal Ca^2+^ signaling has been implicated as a possible underlying mechanism for ASD [18]. Additionally, mutations in *CACNA1C* (encodes L-type voltage gated calcium channel protein), impaired inositol triphosphate receptor (IP3R; responsible for Ca^2+^ transport), and mutations in presynaptic Ca2 + -activated K + channel (BKCa) have been linked to ASD [18].

Neuroinflammation is another possible risk factor for the development of ASD as it has been observed in several areas of post-mortem brains of patients with ASD. Elevated levels of inflammatory cytokines, anti-brain autoantibodies, and maternal immune dysregulation during pregnancy have been reported in ASD, reflecting immune system dysfunction as a contributing factor to ASD [18]. Cerebellar pathology is another factor underlying the neurological dysfunctions observed in ASD. The cerebellum contributes to motor and cognitive functions, rendering it crucial for neurodevelopment. In post-mortem brains of individuals with ASD, immune dysregulation and abnormal cytokine alterations were identified in cerebellar specimen, contributing to cerebellar neuroinflammation. This observation suggests that cerebellar damage and inflammation contributes to ASD pathology [21]. Vitamin D (calcitriol) is a neurosteroid, which has neuroprotective roles, including antiepileptic effects, immunomodulation, and regulation of behaviors in the developing brain, rendering it crucial for normal brain functioning [22]. A systematic review investigating vitamin D deficiency and ASD found the association of significantly lower levels of vitamin D in children with ASD [22]. Similar results were reported in a comprehensive study, in which children with ASD had a mean vitamin D serum level value of 13.79 ± 1.03 ng/ml compared to 16.58 ± 1.06 ng/ml in healthy controls [23], suggesting that vitamin D deficiency contributes to altered brain development in ASD. Lastly, reduced production of indole-3-propionic acid (IPA), a tryptophan metabolite of gut microbiota, due to a dysregulated microbiota-IPA-brain axis has been shown to increase ASD susceptibility. Prenatal antibiotic exposure alters the gut microbiota of infants, potentially contributing to reduced IPA. Also, IPA is a ligand of the aryl hydrocarbon receptor (AHR), which crosses the blood brain barrier and plays a role in immune regulation and inflammation. A recent study reported that fecal microbiota transplantation from children with ASD to germ-free mice induced ASD-like behaviors, suggesting an association between gut microbiota and ASD. Another study demonstrated that microglial hyperactivation in ASD patients could be due to the deletion of AHR. These findings suggest that a disrupted gut microbiota and downregulation of the IPA-AHR pathway contribute to ASD pathophysiology, emphasizing the role of the gut-brain axis [20, 24].

There is increasing evidence that individuals with ASD exhibit a distinct metabolic profile due to disruptions in metabolic pathways. A study investigating the metabolic patterns within three ASD sub-groups exhibiting distinct phenotype patterns unveiled abnormal lipid metabolism pathways such as decreased lipid metabolism associated with increases in amino acid pathways, increased lipid oxidation products, and increased sphingolipid and fatty acid byproducts. This suggests that variations in metabolic pathways underlie the different phenotypes and characteristics observed in ASD [25]. Furthermore, environmental factors, such as dietary habits and sleep disturbances, have impact on the metabolic profiles of individuals with ASD. A study examining the interplay between environmental factors and the plasma lipidome in children found that poor diet and sleep disturbances impact the lipidome profile, gut microbiome, and metabolism in similar ways, potentially leading to long-lasting impacts on the health of children with ASD [26]. This highlights the complex interplay between environmental factors, lipidome profiles, and neurodevelopment in ASD pathophysiology.

Fragile X syndrome (FXS) or Martin-Bell syndrome, is a genetic disorder characterized by cognitive impairment, sensory abnormalities, and epileptic seizures [18]. Approximately 1–6% of ASD cases are attributed to FXS as a large proportion of individuals with FXS are co-diagnosed with ASD [18]. FXS is due to a repetitive mutation causing CGG expansion within the *FMR1* gene, leading to the absence of Fragile X mental retardation protein (FMRP) [27]. Mutation in the FMR1 gene and loss of FMRP cause synaptic dysregulation, leading to behavioral and cognitive impairments, learning deficits, and sensory abnormalities associated with ASD [18]. Rett syndrome is a neurodevelopmental disorder leading to neurologic defects and behavioral symptoms similar to ASD [28]. It is due to a point mutation in methyl-CpG-binding protein (MeCP2), which regulates gene expression through transcriptional repression and chromatin organization [18, 28]. Methyl-CpG-binding domain (MBD) is one of the four domains of *MeCP2* and is essential for normal functioning of *MeCP2* [18]. Mutations in this domain have been associated with synaptic and neural defects, leading to cognitive impairment observed in ASD [18]. Furthermore, a study by Wen et al. identified loss of function *MeCP2* mutations associated with Rett syndrome in patients with ASD, suggesting that these mutations may be involved in the development of ASD [29]. While the etiology of ASD is complex and not fully understood, there is increasing evidence demonstrating that its underlying molecular pathways are multifactorial in origin.

### Epigenetics of ASD

Although the underlying molecular mechanisms of ASD remain to be fully understood, there is increasing evidence suggesting the role of epigenetics in the pathogenesis of ASD. The epigenetic mechanism involved in regulating social behavior in ASD includes histone modification [30]. A population-based study examining the risk of ASD and prenatal exposure to antiseizure medications found that the incidence of ASD was 4.2% in children not exposed to antiseizure medications, 6.2% in children exposed to topiramate, 10% in children exposed to valproate, and 4.1% in children exposed to lamotrigine, suggesting an increased risk of ASD with prenatal exposure to valproate compared to other antiseizure medications [31]. Oral contraceptive use has also been proposed as an epigenetic factor potentially contributing to the risk of developing ASD owing to neurodevelopmental effects [32]. Ethinylestradiol (EE2) is a synthetic hormone in oral contraceptives, which is an endocrine disrupting compound (EDC) that inhibits pregnancy. It is hypothesized that EE2 causes DNA methylation of the estrogen receptor beta gene (ERβ), leading to decreased mRNA of estrogen receptor beta and contributing to developmental effects and impaired brain estrogen signaling [32]. Variation in diet, specifically folic acid consumption, has been implicated to contribute to ASD development [33]. Methylenetetrahydrofolate reductase (MTHFR), an enzyme coded by the gene *MTHFR*, is essential for folate metabolism and DNA, RNA, and protein synthesis [34]. Polymorphisms in *MTHFR*, specifically the C677T polymorphism, have been linked to an increased risk for developing neurological disorders such as ASD. In a meta-analysis comparing the prevalence of ASD in children from countries with and without a folate acid fortified diet, there was a significant association of the MTHFR C677T polymorphism in children from countries without a folate acid fortified diet [33]. These studies suggest that epigenetic factors play an important role in the predisposition to ASD.

## Potential therapeutic targets

### Common molecular pathways

**Fig. 3:**
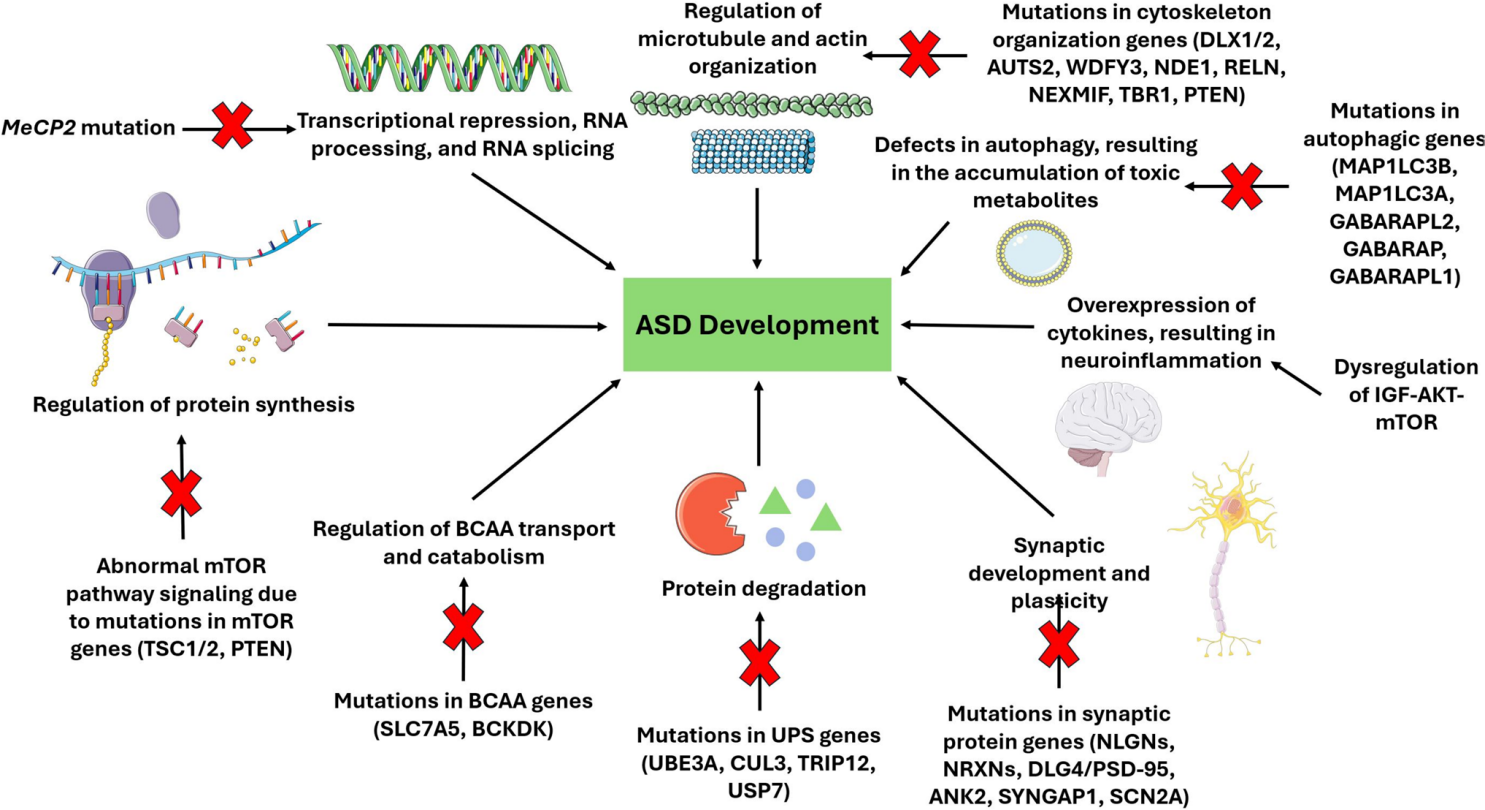
Common Molecular Pathways involved in ASD. This illustration demonstrates several common molecular pathways involved in the pathogenesis of ASD. Mutations in mTOR, BCAA, UPS, synaptic protein, cytoskeleton organization, and autophagic genes can lead to ASD development. *MeCP2* mutations and the dysregulation of the IGF-AKT-mTOR pathway can also lead to ASD pathogenicity [35–37, 64, 65]. This figure was created using Microsoft PowerPoint and Smart Servier Medical Art

Because ASD has such high genetic variability, it has been difficult to establish a broad spectrum treatment. Despite decades of clinical trials, there are currently no approved medications to treat the core symptoms of ASD [35]. Due to the large number of genes, chromosomal abnormalities, and mutations that are associated with ASD, researchers have suggested the categorization of ASD genes into subtypes based on their common molecular pathways, including neurogenesis, synaptogenesis and synaptic plasticity, and transcription and translation [36] (Table 1, Fig. 3). A possible ASD subtype includes genes involved in transcriptional regulation. These genes may act as transcription factors, chromatin modifiers, or regulators of DNA and histone modifications. A highly studied transcriptional regulator associated with the symptoms of ASD is *MECP2*, which plays a role in transcriptional repression and RNA processing and splicing [36]. Researchers have found that the neurological and behavioral phenotypes associated with MeCP2 mutations are reversible, suggesting *MeCP2* gene therapy as a promising target. However, further research needs to be conducted to determine which cell types to target and the possible side effects and toxicity associated with gene over dosage [36].

**Table 1:** Genes involved in ASD pathogenesis

Gene	Gene Function	Reference
Deleted in autism (*DIA-1*)	The Deleted in Autism (*DIA-1*) gene is ubiquitously expressed and localized to the lumen of the Golgi apparatus. It encodes signal peptides for targeting to the secretory pathway. Although little is known about *DIA-1*, it has been linked to the etiology of autism, autism-like syndromes, and mental retardation	[18, 41]
Protocadherin (*PCDH*)	Protocadherin (*PCDH*) is predominantly expressed in the nervous system and is the largest subfamily within the cadherin family. PCDHs are a group of cell adhesion molecules that serve multiple functions within our nervous system, including dendrite development and neural circuit formation	[18, 42]
Methyl CpG Binding Protein 2 (*MeCP2*)	Methyl CpG Binding Protein 2 (*MeCP2*) is known to function as a transcriptional repressor. It inhibits gene expression through DNA methylation and histone acetylation. Mutations in *MeCP2* have been linked to neurodevelopmental disorders such as Rett syndrome	[36, 43]
Tuberous Sclerosis Complex (*TSC1/2*) Phosphatase and Tensin Homolog (*PTEN*) Neurofibromin 1 (*NF1*)	**Tumor Suppressor Genes:** *TSC1/2* genes inhibit the mTOR complex 1 pathway, and together, the TSC/mTORC1 pathway play an integral role in neuronal development pathways such as maturation, synaptic plasticity, and myelination Phosphatase and Tensin Homolog (*PTEN*) is a tumor suppressor gene with protein and lipid phosphatase activity. *PTEN* has been linked to the pathogenesis and prognosis of many cancers, as it plays a role in the regulation of the cell cycle, inducing apoptosis, and inhibiting cell invasion Neurofibromin 1 (*NF1*) is a tumor suppressor gene involved in several cell signaling pathways including Ras/MAPK and Akt/mTOR. *NF1* also regulates many cellular processes such as proliferation, cytoskeletal dynamics, and dopamine levels	[18, 36, 44–46]
Fragile X Messenger Ribonucleoprotein 1 (*FMR1*)	Fragile X Messenger Ribonucleoprotein 1 (*FMR1*) is a RNA binding protein and translator repressor protein. Mutated *FMR1* results in excessive protein synthesis in the brain, which impairs higher cognitive function	[47]
Ubiquitin Protein Ligase E3A (*UBE3A*) Cullin-3 (*CUL3*) Thyroid Hormone Receptor Interactor 12 (*TRIP12*) Ubiquitin-Specific-Processing Protease 7 (*USP7*)	**Protein Degradation:** Ubiquitin Protein Ligase E3A (*UBE3A*) is a gene involved in the ubiquitin protein degradation system. Mutations in *UBE3A* have been linked to neurodevelopmental disorders such as ASD and Angelman Syndrome Cullin-3 (*CUL3*) belongs to a family of ubiquitin ligases. It is involved in a variety of cellular processes including cell division, differentiation, cytoskeleton remodeling, and stress responses Thyroid Hormone Receptor Interactor 12 (*TRIP12*) encodes a ubiquitin ligase and is involved in the regulation of the cell cycle, DNA damage repair, chromatin remodeling, and cell differentiation Ubiquitin-Specific-Processing Protease 7 (*USP7*) is the largest family of deubiquitinating enzymes (DUBs). DUBs reverse and antagonize the effect of ubiquitination. *USP7* also regulates several cellular processes including DNA damage and repair, immune responses, and tumor progression	[36, 48–51]
Solute Carrier Family 7 Member 5 (*SLC7A5*) Branched Chain Keto Acid Dehydrogenase Kinase (*BCKDK*)	**Regulation of Branched Chain Amino Acids (BCCA):** Solute Carrier Family 7 Member 5 (*SLC7A5*) regulates branched chain amino acid (BCAA) transport. Specifically, it is known to be a large neutral amino acid transporter 1 (*LAT1*) and transports amino acids such as leucine, tyrosine, and tryptophan, which are crucial to biological functions. *SLC7A5* is also involved in the Mechanistic Target of Rapamycin Kinase Complex 1 (mTORC1), which is a nutrient signaling pathway that regulates cellular metabolism Branched Chain Keto Acid Dehydrogenase Kinase (*BCKDK*) functions as a rate-limiting enzyme in branched chain amino acid (BCAA) metabolism. *BCKDK* codes for a kinase that downregulates the activity of branched chain alpha-keto acid dehydrogenase complex (BCKD), preventing the catabolism of BCAAs	[36, 52–54]
Activator of Transcription and Developmental Regulator 2 (*AUTS2*) WDFY3 NudE Neurodevelopment Protein 1 (*NDE1*) Reelin (*RELN*) Neurite Extension and Migration Factor (*NEXMIF*) T-Box Brain Transcription Factor 1 (*TBR1*)	**Neurodevelopment:** Activator of Transcription and Developmental Regulator 2 (*AUTS2*) is a gene involved in various neurodevelopmental pathways. These pathways include regulating transcription in cell nuclei, neuronal migration in cytoplasm, and cytoskeletal organization *WDFY3* plays a role in neurodevelopmental processes, cognitive functions, cerebral cortical size regulation, and prenatal neurogenesis NudE Neurodevelopment Protein 1 (*NDE1*) plays a role in cortical development and migration of glial cells, which are crucial for adequate neurodevelopment and cognitive capabilities Reelin (*RELN*) is a large extracellular protein that has multiple important functions during embryonic development such as neocortical neuronal migration, synaptic plasticity, dendritic growth, and development of the dendritic spine Neurite Extension and Migration Factor (*NEXMIF*) is a gene expressed in the cerebral cortex and cerebellum. It encodes the neurite growth-directed factor that is important for neurite extension and migration and is crucial for nerve development T-Box Brain Transcription Factor 1 (*TBR1*) is known to be involved in the development of the cerebral cortex and the amygdala as well as regulation of neuronal migration and axonal projection	[18, 36, 55–60]
Neuroligins (*NLGs*) β-neurexins (*NRXNs*) Discs Large MAGUK Scaffold Protein 4 (*DLG4*) Postsynaptic Density Protein 95 (*PSD-95*) Ankyrin 2 (*ANK2*) Synaptic Ras GTPase-activating protein 1 (*SYNGAP1*) Sodium Voltage-Gated Channel Alpha Subunit 2 (*SCN2*) SH3 and Multiple Ankyrin Repeat Domains 3 (*SHANK3*)	**Synaptic development and plasticity:** Neuroligins (*NLGs*) and β-neurexins (*NRXNs*) are two genes that play important roles in the process of neural synapses and synaptic cell adhesion via stimulation of the cholinergic and glutaminergic neural synaptogenesis as well as regulation of other physiological and learning functions Discs Large MAGUK Scaffold Protein 4 (*DLG4*) is a gene that encodes Postsynaptic Density Protein 95 (*PSD-95*), which is a major regulator of synaptic maturation by interacting, stabilizing, and trafficking N-methyl-D-aspartic acid receptors and amino-3-hydroxy-5-methyl-4-isox-azoleproprionic acid receptors to the postsynaptic membrane Ankyrin 2 (*ANK2*) is a gene that encodes for a cytoskeletal scaffolding protein involved in recruiting membrane proteins into specialized membrane domains and regulating neural stem cell differentiation and neuronal migration in the embryonic cerebral cortex Synaptic Ras GTPase-activating protein 1 (*SYNGAP1*) is a gene encoding a synaptic Ras GTPase-activating protein that localizes to dendritic cells in neocortical pyramidal neurons, suppressing NMDA receptor-mediated synaptic plasticity Sodium Voltage-Gated Channel Alpha Subunit 2 (*SCN2*) is a gene that encodes the Na + channel proteins NaV_1.1_ and NaV_1.2_, both of which play key roles in autism symptoms, such as seizure and epilepsy SH3 and Multiple Ankyrin Repeat Domains 3 (*SHANK3*) is a gene that primarily regulates dendritic spine maintenance and maturation. They are involved in glutamate receptor-mediated neuronal signaling and promote the scaffolding of glutamate receptor polypeptides and the release of Ca^2+^ ions from the ER	[18, 36, 61–64]
Microtubule Associated Protein 1 Light Chain 3 Beta (*MAP1LC3B*) Microtubule Associated Protein 1 Light Chain 3 Alpha (*MAP1LC3A*) Gamma-Aminobutyric Acid Receptor-Associated Protein-Like 2 (*GABARAPL2*) Gamma-Aminobutyric Acid Receptor-Associated Protein (*GABARAP*) GABA Type A Receptor Associated Protein Like 1 (*GABARAPL1*)	**Autophagy:** Microtubule Associated Protein 1 Light Chain 3 Beta (*MAP1LC3B*) and Microtubule Associated Protein 1 Light Chain 3 Alpha (*MAP1LC3A*) encode for a subunit of neuronal microtubule-associated MAP1B and MAP1A proteins, which are involved in microtubule assembly and important for neurogenesis Gamma-Aminobutyric Acid Receptor-Associated Protein-Like 2 (*GABARAPL2*) and GABA Type A Receptor Associated Protein Like 1 (*GABARAPL1*) enables ubiquitin protein ligase binding activity and is predicted to be involved in several processes, including autophagosome assembly, autophagy of mitochondria, and negative regulation of proteasomal protein catabolic process Gamma-Aminobutyric Acid Receptor-Associated Protein (*GABARAP*) is a gene that encodes a protein that is associated with ligand-gated chloride channels that mediate inhibitory neurotransmission. The protein clusters neurotransmitter receptors by mediating interaction with the cytoskeleton	[65]

This table illustrates the variety of genes associated with ASD and their specific functions

Proteostasis is another common molecular pathway linked to ASD. Protein synthesis is regulated by various cascades, including the mammalian target of rapamycin (mTOR) and FMRP, whereas protein degradation is regulated by the ubiquitin-protease system (UPS). Several genes involved in protein synthesis (*TSC1/2, PTEN, NF1, FMR1*) and degradation (*UBE3A, CUL3*) have been linked to ASD phenotypes [36]. The mTOR pathway integrates multiple intracellular and extracellular signals to coordinate cellular responses including protein synthesis and growth [37]. mTOR is a member of two protein families: mTORC1 and mTORC2. mTORC1 positively regulates anabolic processes and negatively regulates catabolic processes, whereas mTORC2 controls downstream protein kinases. Researchers believe that overactivation of mTORC1 is responsible for the symptoms of ASD and other related neurodevelopmental disorders [37]. In animal model studies of tuberous sclerosis, inhibition of the mTOR pathway in young animals prevented neurological and behavioral phenotypes. In adult animals, however, manipulation of the mTOR pathway was less successful in preventing these pathogenic phenotypes [36]. In another trial, the use of everolimus, an mTOR inhibitor, on intellectual disability in children with tuberous sclerosis complex (TSC) revealed minimal improvement in cognitive functioning or other neuropsychological deficits in children with TSC [38]. Although the mTOR pathway seemed like a promising target, mTOR inhibitors have fallen short of improving neurocognitive symptoms.

Another aspect of proteostasis that has been linked to ASD is the regulation of branched chain amino acid (BCAA) levels. Studies have found that homozygous loss-of-function mutations in *SLC7A5* and *BCKDK*, genes involved in the regulation of BCAA transport and catabolism, respectively, are associated with ASD. Additionally, multiple studies have found links between ASD and protein degradation defects within the ubiquitin proteasome system (UPS), including *UBE3A, CUL3, TRIP12* and *USP7* [36]. From these findings, we see that protein homeostasis may play a central role in the development of ASD symptoms, thereby serving as a promising avenue for treatment.

Another possible ASD subtype is genes involved in cytoskeletal organization, such as microtubule and actin organization. Several ASD genes have been associated with the regulation of cytoskeleton organization: *DLX1/2, AUTS2, WDFY3, NDE1, RELN, NEXMIF, TBR1,* and *PTEN*. Although the development of treatments targeting genes involved in cytoskeleton organization has been limited, potential treatments may utilize postnatal gene re-expression and early genetic testing allowing for the development of embryonic treatments [36].

Another common molecular pathway linked to ASD symptoms is synaptic development and plasticity, including cell adhesion and postsynaptic density proteins. Genes encoding synaptic proteins (*NLGNs, NRXNs, DLG4/PSD-95, ANK2, SYNGAP1* and *SCN2A*) were among the first to be identified as having a correlation with ASD [36]. Synaptic development and plasticity are strongly associated with other molecular pathways discussed here, including protein synthesis and cytoskeleton organization. Therefore, successful treatments may need to simultaneously target multiple molecular pathways. As discussed earlier, Fragile X syndrome (FXS) is a disorder associated with ASD and is due to a mutation in the FMR1 gene, leading to the loss of the FMRP protein, resulting in synaptic dysregulation. An abnormal increase of metabotropic glutamate receptors (mGluRs) is a key feature of FXS. mGluRs play a role in regulating the response to glutamate via long-term potentiation and long-term depression, which are crucial processes for synaptic plasticity. This finding led to the formulation of the mGluR theory, which states that the loss of FMRP leads to mGluR type I activation, enhancing long-term depression and decreasing long-term potentiation, resulting in the cognitive impairments observed in FXS. This suggests that mGluR activity may be a potential treatment target to address the synaptic dysregulation associated with FXS [39]. However, multiple clinical trials aimed at decreasing mGluR signaling with negative allosteric modulators (NAMs) to correct synaptic plasticity exhibited inconsistent outcome [40]. This highlights that identifying ASD-related genes and the research regarding the impact of these genes in the pathogenic pathways of ASD is crucial.

### Autophagy

Autophagy involves the degradation of intracellular substances and organelles via lysosomes and these degradation products serve as energy sources for other cellular processes. It is induced by toxic, metabolic, environmental, oxidative, and inflammatory stress. While it is well known that autophagy plays a central role in the maintenance of homeostasis and adaptive responses in the brain, more recent studies have suggested that autophagy is involved in the production of new neurons and renewal of neural stem cells (NSCs). Therefore, defects in autophagy underlie several neurodevelopmental disorders, including ASD [65]. Altered autophagy results in the accumulation of toxic metabolites, making NSCs more susceptible to damage. Studies have identified multiple autophagic genes that have been deleted in ASD: *MAP1LC3B, MAP1LC3A, GABARAPL2, GABARAP,* and *GABARAPL1*. Furthermore, other non-autophagic ASD-related genes, including *TSC1, TSC2, mTOR,* and *PTEN*, are directly linked to the regulation of autophagy and subsequent NSC development and differentiation [65]. Although researchers have explored treatments targeting autophagic genes, these studies have yet to yield sufficient evidence of success. However, with direct correlations to ASD pathogenesis, autophagy remains a promising therapeutic target to be further explored.

### Insulin-like growth factors

The role of insulin-like growth factors (IGF-1) in ASD has been widely explored and researchers have observed that there is dysregulation of the IGF-AKT-mTOR axis in ASD. IGF-1 is a naturally occurring protein with important functions in brain development. IGF-1 triggers the transcription of proteins involved in synaptic development through the MAPK and mTOR pathways [35]. Neuroinflammation is a common component of ASD and is caused by the overexpression of inflammatory cytokines in the brain, such as IL-6. IGF-1 reduces neuroinflammation and regulates IL-6 expression [64]. IGF-1 is currently being tested in clinical trials conducted on subjects with Rett syndrome, Fragile X syndrome, and *SHANK3* deficiency. In one set of trials, two different IGF-1 peptides are being utilized: recombinant human IGF-1 and a newer drug, IGF-1(35,36,45). IGF-1(35,36,45) can cross the blood–brain barrier, can be administered orally, and does not bind with IGF-1 receptors, thereby reducing its mitogenic properties [64]. In another set of clinical trials, researchers administered IGF-1 to patients with *SHANK3* mutations and observed significant improvements in social impairment and restrictive behaviors [36]. IGF-1 analogs, such Trofinetide, were tested in clinical trials for the treatment of Rett Syndrome. Trofinetide is a synthetic analog of glycine-proline-glutamate (GPE), which is a naturally occurring N-terminal tripeptide in the brain that is cleaved from IGF-1 [66]. Trofinetide treatment significantly improved the core symptoms of Rett Syndrome such as cognitive function, communication, motor skills, and behavioral skills [66]. In addition to these human trials, IGF-1 has been studied in MECP2 knockout mouse models of Rett Syndrome. In these mouse models, IGF-1 increased dendritic spine density, reduced cytokine expression and neuroinflammation, and activated the AKT-mTOR pathway in microglia [64]. Overall, the findings from animal models and human trials hail IGF-1 to be a promising target for ASD management.

### Advances in ASD research

Since the mid-twentieth century, research methods and paradigms regarding ASD have undergone extensive evolution. In its early years, ASD was mistaken for an emotional disorder that formed the basis of research that focused primarily on psychoanalysis and behavioral observations. With remarkable advancements in neurogenetics in the second half of the twentieth century, a shift began that emphasized the genetic factors and abnormalities in brain structure and function [67]. Also, a large number of twin and family studies that discovered these genetic predispositions to ASD. In addition, neuroimaging studies uncovered specific brain development abnormalities. These changes marked the important shift in research from the psychosocial model to the biomedical model. Current research exploits bioinformatics, high-throughput gene sequencing technology, and computational modeling to identify specific genetic variants, integrate epigenetics, and incorporate new perspectives in ASD research [67].

Emerging knowledge in ASD research paved the way for advanced ASD diagnostic approaches. Traditionally, ASD diagnosis relied heavily on clinical assessments of behavior and development that are easily plagued by subjective inaccuracies and discrepancies. Such approaches create diagnoses that are variable, and inaccurate. Three important diagnostic techniques have emerged to address this subjectivity. The first includes genetic testing that looks at specific gene mutations that have been shown to increase ASD risk [67]. The second method includes neuroimaging, which includes “functional magnetic resonance imaging (fMRI), structural magnetic resonance imaging (sMRI), diffusion tensor imaging (DTI), and positron emission tomography (PET)”, in order to observe structural and electrochemical activity in those with ASD [67]. The last technique is early screening methods, which have recently been leveraging artificial intelligence and machine learning in order to recognize specific patterns of behaviors in children such as eye movement patterns using eye-tracking technology [68]. With further research and refinement of these techniques, the efficacy and accuracy of ASD diagnosis is expected to be improved.

### Translational outcomes and future research

A wide range of genes and molecular pathways are implicated in the development of ASD. However, since most cases of ASD are idiopathic and the etiology is highly complex, it is difficult to develop targeted treatments. Although therapeutic interventions for ASD are limited, there are a few therapies currently available, including behavioral therapies, diet interventions, and pharmacological treatments. While these treatments cannot reverse ASD completely and depend on the severity of symptoms, they seek to improve quality of life, skills, abilities, and reduce symptoms [69].

Behavior-oriented interventions aim to recondition target behaviors, improve daily living competencies, and address social, communicative, and cognitive skills. A widely used behavioral treatment is applied behavior analysis (ABA), which is based on the principles of learning and operant conditioning [70]. ABA aims to recondition target behaviors by teaching specific skills and behaviors through positive reinforcement [69]. Positive reinforcement is a key principle of this intervention because when a behavior is followed by a reward, the behavior is most likely to be repeated. The goal of this intervention is to reduce inappropriate behavior and increase socially appropriate behaviors [70]. A meta-analysis that investigated the efficacy of ABA-based interventions in the treatment of children with ASD, found that ABA-based treatments resulted in improvements in intellectual functioning and adaptive behavior [71]. Dietary intervention is another approach to treat ASD through incorporation of vitamins and minerals such as vitamin D supplementation, especially during pregnancy and early childhood. This intervention supports normal and healthy development of the brain with improved function. Additionally, treatment with vitamin B6, vitamin C, magnesium, and omega-3 fatty acid supplementation led to behavioral improvements in children with ASD [69].

Pharmacological treatments are commonly used to treat individuals with ASD. The most prescribed drugs are Abilify (aripiprazole) and Risperdal (risperidone) [69]. Although these drugs are FDA approved, they were not originally developed to treat ASD but have been shown to target specific ASD symptoms. For example, aripiprazole is an antipsychotic medication, but has been used to treat aggression, social withdrawal, self-injurious behavior, hyperactivity, stereotypies, and sleep disturbances. Additionally, melatonin can be used to treat sleep disturbances and valproic acid can be used to treat mood swings, bipolar disorder, and seizures in people with ASD [69]. Preclinical and clinical trials are currently investigating the efficacy of other drugs in treating ASD symptoms. Preliminary research in animal models has shown that ampakines, specifically ampakines CX1837 and CX1739, improve learning, memory, and social behaviors [69]. While these pharmacotherapy treatments address common ASD symptoms, future research to develop drugs that target the core underlying molecular pathways and genes involved in ASD, is warranted.

Historically, in vivo animal models have been the primary tool used to study early brain development and neurodevelopmental disorders including ASD. More recently, in vitro human models have been created using human neural precursor cells (phNPCs) from the fetal postmortem cortex by the induction of neurons from non-neuronal cells, and embryonic (ESC) or induced pluripotent stem cells (iPSC) and 2D or 3D neuronal cultures [72]. In vitro human models provide human-specific insights, higher genetic specificity, and cellular-level detail that in vivo animal models often fail to achieve. However, such models lack the brain complexity and genetic heterogeneity. A model based on a single genetic mutation in ASD (for example, a *SHANK3* deficiency) fails to represent the diversity of genetic factors contributing to ASD pathogenesis [72]. In vivo animal models still serve as a critical tool for understanding ASD; however, in vitro human models advance and evolve with time to provide a more comprehensive picture of ASD, possibly surpassing the utility of traditional animal models.

## Conclusion

ASD is a complex, lifelong condition that involves various genes and molecular mechanisms. Although there are multiple molecular mechanisms and genes implicated in ASD pathogenesis, *MeCP2* and insulin-like growth factor (IGF-1) appear to be the most promising targets for therapeutic intervention due to their strong association with ASD, as well as abundance of research and clinical trials supporting their potential efficacy. Because *MECP2* mutations have underlying synaptic and neural defects that are reversible, *MeCP2* gene therapy should be further explored as a treatment for ASD. Furthermore, clinical trials have successfully proven that treatment with IGF-1 can help improve the social deficits seen in ASD patients, which warrants further research for the use of IGF-1 as a therapeutic intervention for ASD. Moving forward, further research is needed to explore how the molecular pathways, genes, and biomarkers associated with ASD can be effectively translated into targeted therapeutic strategies.

## Data Availability

No datasets were generated or analysed during the current study.
